# HLA-DRB1 and HLA-DQB1 genetic diversity modulates response to lithium in bipolar affective disorders

**DOI:** 10.1038/s41598-021-97140-7

**Published:** 2021-09-08

**Authors:** Sigrid Le Clerc, Laura Lombardi, Bernhard T. Baune, Azmeraw T. Amare, Klaus Oliver Schubert, Liping Hou, Scott R. Clark, Sergi Papiol, Micah Cearns, Urs Heilbronner, Franziska Degenhardt, Fasil Tekola-Ayele, Yi-Hsiang Hsu, Tatyana Shekhtman, Mazda Adli, Nirmala Akula, Kazufumi Akiyama, Raffaella Ardau, Bárbara Arias, Jean-Michel Aubry, Lena Backlund, Abesh Kumar Bhattacharjee, Frank Bellivier, Antonio Benabarre, Susanne Bengesser, Joanna M. Biernacka, Armin Birner, Clara Brichant-Petitjean, Pablo Cervantes, Hsi-Chung Chen, Caterina Chillotti, Sven Cichon, Cristiana Cruceanu, Piotr M. Czerski, Nina Dalkner, Alexandre Dayer, Maria Del Zompo, J. Raymond DePaulo, Bruno Étain, Stephane Jamain, Peter Falkai, Andreas J. Forstner, Louise Frisen, Mark A. Frye, Janice M. Fullerton, Sébastien Gard, Julie S. Garnham, Fernando S. Goes, Maria Grigoroiu-Serbanescu, Paul Grof, Ryota Hashimoto, Joanna Hauser, Stefan Herms, Per Hoffmann, Esther Jiménez, Jean-Pierre Kahn, Layla Kassem, Po-Hsiu Kuo, Tadafumi Kato, John R. Kelsoe, Sarah Kittel-Schneider, Ewa Ferensztajn-Rochowiak, Barbara König, Ichiro Kusumi, Gonzalo Laje, Mikael Landén, Catharina Lavebratt, Susan G. Leckband, Alfonso Tortorella, Mirko Manchia, Lina Martinsson, Michael J. McCarthy, Susan L. McElroy, Francesc Colom, Vincent Millischer, Marina Mitjans, Francis M. Mondimore, Palmiero Monteleone, Caroline M. Nievergelt, Markus M. Nöthen, Tomas Novák, Claire O’Donovan, Norio Ozaki, Urban Ösby, Andrea Pfennig, James B. Potash, Andreas Reif, Eva Reininghaus, Guy A. Rouleau, Janusz K. Rybakowski, Martin Schalling, Peter R. Schofield, Barbara W. Schweizer, Giovanni Severino, Paul D. Shilling, Katzutaka Shimoda, Christian Simhandl, Claire M. Slaney, Claudia Pisanu, Alessio Squassina, Thomas Stamm, Pavla Stopkova, Mario Maj, Gustavo Turecki, Eduard Vieta, Julia Veeh, Stephanie H. Witt, Adam Wright, Peter P. Zandi, Philip B. Mitchell, Michael Bauer, Martin Alda, Marcella Rietschel, Francis J. McMahon, Thomas G. Schulze, Jean-Louis Spadoni, Wahid Boukouaci, Jean-Romain Richard, Philippe Le Corvoisier, Caroline Barrau, Jean-François Zagury, Marion Leboyer, Ryad Tamouza

**Affiliations:** 1 Laboratoire Génomique, Bio-Informatique et Chimie Moléculaire (EA7528), Conservatoire National des Arts et Métiers, HESAM Université, 292, rue Saint Martin, 75003 Paris, France; 2 AP-HP, Hôpital Henri Mondor, Département Médico-Universitaire de Psychiatrie et D’Addictologie (DMU IMPACT), Fédération Hospitalo-Universitaire de Médecine de Précision (FHU ADAPT), 94010 Créteil, France; 3 INSERM U955, IMRB, Laboratoire Neuro-Psychiatrie Translationnelle, Université Paris Est Créteil, 94010 Créteil, France; 4 Fondation FondaMental, Créteil, France; 5 Department of Psychiatry, University of Münster, Münster, Germany; 6 Department of Psychiatry, Melbourne Medical School, The University of Melbourne, Melbourne, Australia; 7 The Florey Institute of Neuroscience and Mental Health, The University of Melbourne Parkville, Parkville, VIC Australia; 8 Discipline of Psychiatry, School of Medicine, University of Adelaide, Adelaide, SA Australia; 9 South Australian Academic Health Science and Translation Centre, South Australian Health and Medical Research Institute (SAHMRI), Adelaide, SA Australia; 10 Mental Health Services, Northern Adelaide Local Health Network, Adelaide, SA Australia; 11 Intramural Research Program, National Institute of Mental Health, National Institutes of Health, US Department of Health & Human Services, Bethesda, MD USA; 12 Institute of Psychiatric Phenomics and Genomics (IPPG), University Hospital, LMU Munich, Nußbaumstr. 7, 80336 Munich, Germany; 13 Department of Psychiatry and Psychotherapy, Ludwig-Maximilian-University Munich, Munich, Germany; 14 Institute of Human Genetics, University of Bonn, School of Medicine & University Hospital Bonn, Bonn, Germany; 15 Epidemiology Branch, Division of Intramural Population Health Research, Eunice Kennedy Shriver National Institute of Child Health and Human Development, National Institutes of Health, Bethesda, MD USA; 16 HSL Institute for Aging Research, Harvard Medical School, Boston, MA USA; 17 Program for Quantitative Genomics, Harvard School of Public Health, Boston, MA USA; 18 Department of Psychiatry, University of California San Diego, La Jolla, CA USA; 19 Department of Psychiatry and Psychotherapy, Charité - Universitätsmedizin Berlin, Campus Charité Mitte, Berlin, Germany; 20 Department of Biological Psychiatry and Neuroscience, Dokkyo Medical University School of Medicine, Mibu, Tochigi, Japan; 21 Unit of Clinical Pharmacology, Hospital University Agency of Cagliari, Cagliari, Italy; 22 Unitat de Zoologia I Antropologia Biològica (Dpt. Biologia Evolutiva, Ecologia I Ciències Ambientals), Facultat de Biologia and Institut de Biomedicina (IBUB), University of Barcelona, CIBERSAM, Barcelona, Spain; 23 Department of Psychiatry, Mood Disorders Unit, HUG - Geneva University Hospitals, Geneva, Switzerland; 24 Department of Molecular Medicine and Surgery, Karolinska Institute, Stockholm, Sweden; 25 Center for Molecular Medicine, Karolinska University Hospital, Stockholm, Sweden; 26 INSERM UMR-S 1144, Département de Psychiatrie et de Médecine Addictologique, AP-HP, Groupe Hospitalier Saint-Louis-Lariboisière-F.Widal, Université Paris Diderot, Paris, France; 27 Bipolar Disorder Program, Institute of Neuroscience, Hospital Clinic, University of Barcelona, IDIBAPS, CIBERSAM, Barcelona, Catalonia Spain; 28 Department of Psychiatry and Psychotherapeutic Medicine, Research Unit for Bipolar Affective Disorder, Medical University of Graz, Graz, Austria; 29 Department of Health Sciences Research, Mayo Clinic, Rochester, MN USA; 30 Department of Psychiatry and Psychology, Mayo Clinic, Rochester, MN USA; 31 The Neuromodulation Unit, McGill University Health Centre, Montreal, QC Canada; 32 Department of Psychiatry & Center of Sleep Disorders, National Taiwan University Hospital, Taipei, Taiwan; 33 Institute of Medical Genetics and Pathology, University Hospital Basel, Basel, Switzerland; 34 Institute of Neuroscience and Medicine (INM-1), Research Center Jülich, Jülich, Germany; 35 Human Genomics Research Group, Department of Biomedicine, University Hospital Basel, Basel, Switzerland; 36 Douglas Mental Health University Institute, McGill University, Montreal, QC Canada; 37 Psychiatric Genetic Unit, Poznan University of Medical Sciences, Poznan, Poland; 38 Department of Biomedical Sciences, University of Cagliari, Cagliari, Italy; 39 Department of Psychiatry and Behavioral Sciences, Johns Hopkins University, Baltimore, MD USA; 40 Univ Paris Est Créteil, INSERM, IMRB, Translational Neuropsychiatry, Fondation FondaMental, Créteil, France; 41 Centre for Human Genetics, University of Marburg, Marburg, Germany; 42 Neuroscience Research Australia, Sydney, NSW Australia; 43 School of Medical Sciences, University of New South Wales, Sydney, NSW Australia; 44 Service de Psychiatrie, Hôpital Charles Perrens, Bordeaux, France; 45 Department of Psychiatry, Dalhousie University, Halifax, NS Canada; 46 Biometric Psychiatric Genetics Research Unit, Alexandru Obregia Clinical Psychiatric Hospital, Bucharest, Romania; 47 Mood Disorders Center of Ottawa, Ottawa, ON Canada; 48 Department of Pathology of Mental Diseases, National Institute of Mental Health, National Center of Neurology and Psychiatry, Tokyo, Japan; 49 Department of Psychiatry, Osaka University Graduate School of Medicine, Osaka, Japan; 50 Service de Psychiatrie Et Psychologie Clinique, Centre Psychothérapique de Nancy - Université de Lorraine, Nancy, France; 51 Department of Public Health & Institute of Epidemiology and Preventive Medicine, College of Public Health, National Taiwan University, Taipei, Taiwan; 52 Department of Psychiatry and Behavioral Science, Juntendo University Graduate School of Medicine, Tokyo, Japan; 53 Department of Psychiatry, Psychosomatic Medicine and Psychotherapy, University Hospital Frankfurt, Frankfurt, Germany; 54 Department of Adult Psychiatry, Poznan University of Medical Sciences, Poznan, Poland; 55 Department of Psychiatry and Psychotherapeutic Medicine, Landesklinikum Neunkirchen, Neunkirchen, Austria; 56 Department of Psychiatry, Hokkaido University Graduate School of Medicine, Sapporo, Japan; 57 Institute of Neuroscience and Physiology, The Sahlgrenska Academy at the Gothenburg University, Gothenburg, Sweden; 58 Department of Medical Epidemiology and Biostatistics, Karolinska Institutet, Stockholm, Sweden; 59 Office of Mental Health, VA San Diego Healthcare System, San Diego, CA USA; 60 Department of Psychiatry, University of Perugia, Perugia, Italy; 61 Section of Psychiatry, Department of Medical Sciences and Public Health, University of Cagliari, Cagliari, Italy; 62 Department of Pharmacology, Dalhousie University, Halifax, NS Canada; 63 Department of Clinical Neurosciences, Karolinska Institutet, Stockholm, Sweden; 64 Department of Psychiatry, VA San Diego Healthcare System, San Diego, CA USA; 65 Department of Psychiatry, Lindner Center of Hope/University of Cincinnati, Mason, OH USA; 66 Mental Health Research Group, IMIM-Hospital del Mar, Barcelona, Catalonia Spain; 67 Centro de Investigación Biomédica en Red de Salud Mental (CIBERSAM), Instituto de Salud Carlos III, Madrid, Spain; 68 Departament de Genètica, Microbiologia i Estadística, Facultat de Biologia, Universitat de Barcelona, Barcelona, Spain; 69 Institut de Biomedicina de la Universitat de Barcelona (IBUB), Barcelona, Spain; 70 Centro de Investigación Biomédica en Salud Mental (CIBERSAM), Madrid, Spain; 71 Neurosciences Section, Department of Medicine, Surgery and Dentistry ‘Scuola Medica Salernitana’, University of Salerno, Salerno, Italy; 72 Department of Psychiatry, University of Campania ‘Luigi Vanvitelli’, Naples, Italy; 73 National Institute of Mental Health, Klecany, Czech Republic; 74 Department of Psychiatry & Department of Child and Adolescent Psychiatry, Nagoya University Graduate School of Medicine, Nagoya, Japan; 75 Department of Neurobiology, Care Sciences, and Society, Karolinska Institutet and Center for Molecular Medicine, Karolinska University Hospital, Stockholm, Sweden; 76 Department of Psychiatry and Psychotherapy, Medical Faculty, University Hospital Carl Gustav Carus, Technische Universität Dresden, Dresden, Germany; 77 Montreal Neurological Institute and Hospital, McGill University, Montreal, QC Canada; 78 Department of Psychiatry, Dokkyo Medical University School of Medicine, Mibu, Tochigi Japan; 79 Bipolar Center Wiener Neustadt, Medical Faculty, Sigmund Freud University, Vienna, Austria; 80 Department of Genetic Epidemiology in Psychiatry, Central Institute of Mental Health, Medical Faculty Mannheim, University of Heidelberg, Mannheim, Germany; 81 School of Psychiatry, University of New South Wales, Sydney, NSW Australia; 82 Department of Mental Health, Johns Hopkins Bloomberg School of Public Health, Baltimore, MD USA; 83 Department of Psychiatry and Psychotherapy, University Medical Center (UMG), Georg-August University Göttingen, Göttingen, Germany; 84 Centre Investigation Clinique, CIC Henri Mondor, Université Paris Est Créteil, 94010 Créteil, France; 85 Plateforme de Ressources Biologiques, HU Henri Mondor, 94010 Créteil, France

**Keywords:** Genetics, Psychiatric disorders, Immunology, Immunogenetics

## Abstract

Bipolar affective disorder (BD) is a severe psychiatric illness, for which lithium (Li) is the gold standard for acute and maintenance therapies. The therapeutic response to Li in BD is heterogeneous and reliable biomarkers allowing patients stratification are still needed. A GWAS performed by the International Consortium on Lithium Genetics (ConLiGen) has recently identified genetic markers associated with treatment responses to Li in the human leukocyte antigens (HLA) region. To better understand the molecular mechanisms underlying this association, we have genetically imputed the classical alleles of the *HLA* region in the European patients of the ConLiGen cohort. We found our best signal for amino-acid variants belonging to the HLA-DRB1*11:01 classical allele, associated with a better response to Li (*p* < 1 × 10^−3^; FDR < 0.09 in the recessive model). Alanine or Leucine at position 74 of the HLA-DRB1 heavy chain was associated with a good response while Arginine or Glutamic acid with a poor response. As these variants have been implicated in common inflammatory/autoimmune processes, our findings strongly suggest that HLA-mediated low inflammatory background may contribute to the efficient response to Li in BD patients, while an inflammatory status overriding Li anti-inflammatory properties would favor a weak response.

## Introduction

Bipolar affective disorder (BD) is a severe and often chronic psychiatric illness, characterized by recurrent dysregulation of mood with alternating episodes of mania and depression. BD affects around 48.8 million people^1^ accounting for 9.9 million years of life lived with disability worldwide^1^. All-cause mortality and risk of suicide^2^ are substantially increased in people with the disorder. Both genetic and environmental factors have been identified to contribute to the pathogenesis of BD^3^ but the underlying molecular processes remain poorly understood. Nevertheless, well codified mood stabilizer-based treatments have totally changed BD patient management.

In this context, Lithium (Li) has gained a status as the ‘gold standard’ amongst mood-stabilizers used for both acute- and maintenance therapy in BD^4–6^. Li display strong anti-manic properties^7,8^. Accordingly, it is the only mood stabilizer with protective effects against further episodes of both manic and depressive polarity^6^, making it the most efficient therapeutic option for preventing relapses^9^. Even if Li is recommended as a first-line option for anti-manic and maintenance treatment^10–13^, its routinely use is hampered by several caveats. Firstly, the therapeutic response to Li in BD is highly heterogeneous. In acute mania, about 65% of patients respond at least partially to Li monotherapy while 35% are refractory^14,15^. In maintenance treatment, an efficient long-term response is reported only for approximately 30% of patients^16^, whereas 30% has intermediate outcomes and 30% poor response. Secondly, Li is toxic at high doses and interindividual variation in metabolism requires a stringent monitoring of Li plasma levels^17^. And thirdly, Li has long-term adverse effects including alteration of thyroid and parathyroid functions and an elevated risk of renal failure^5^.

Amongst biological Li modulator markers, genetic variants (i.e., single nucleotide polymorphisms, SNPs^16,18^) and genetic traits (i.e., polygenic risk scores, PRS) have been identified that differentiate Li response groups. We recently reported that BD patients with high genetic load for schizophrenia and major depressive disorders (MDD) are less likely to have favorable Li treatment outcomes than those with lower genetic load of these traits^19,20^. Moreover, we showed in cross-trait meta-GWAS and pathway analyses that genetic signals tagging the major histocompatibility complex (MHC) and pro-inflammatory cytokines including tumor necrosis factor alpha (TNF-α), interleukin 4 (IL-4) and interferon gamma (IFNγ) could have a biological role in Li treatment response in BD^19^. More specifically, the most significant finding of the cross-trait GWAS and the SNP from the post-GWAS functional analyses suggested that the human leukocyte antigen (HLA) cluster which is hosted by the major histocompatibility complex (MHC) region could be implicated in the genetic susceptibility to schizophrenia (SCZ) and Li treatment response^19^. The MHC/HLA region is the most robust immunogenetic finding in SCZ^21^, and could be marking a schizophrenia-type pathogenesis associated with Li non-response. The extensive linkage disequilibrium (LD) in the *HLA* region, the presence of non-*HLA* genes surrounding the cluster and pitfalls in study designs may explain previous inconsistent data concerning *HLA* association with Li responsiveness in BD^22–24^. Nevertheless, the above-mentioned tags of both HLA and pro-inflammatory cytokines reasonably focus attention towards the possible implication of immune processes in responses to Li.

Accordingly and given the role of *HLA* alleles in antigen presentation to downstream effector immune cell subsets, we took advantage of the above-mentioned GWAS^19^ for an in-depth analysis of genetic variants of the *HLA* region in relation to Li treatment response. To this end, we imputed the *HLA* class-I and class-II genetic variants corresponding to HLA amino-acids and classical alleles and investigated their distribution in responders and non-responders to Li treatment.

## Results

Genotyping data from 2139 BD patients who had long-term Li treatment were obtained from the International Consortium ConLiGen for *HLA* imputation (847 in GWAS1 and 1292 in GWAS2). The descriptive statistics of the phenotypes of the participants in each analysis are presented in Table 1. The main goal of the present study was to uncover HLA amino-acids or classical *HLA* alleles potentially associated with responses to Li in BD. We first performed association analyses with *HLA* variants separately for the GWAS1 and GWAS2, and then performed a meta-analysis of the two studies.

**Table 1:** Phenotypic characteristics of individuals used for the analyses.

	GWAS1	GWAS2
**All_individuals**	847	1292
Age at interview	48.15 (13.78)	46.90 (14.01)
**Sex**		
Men	360 (43%)	565 (44%)
Women	487 (57%)	727 (56%)
Alda scale A score	6.14 (3.05)	6.48 (2.77)
Alda scale total B score	2.16 (1.63)	2.86 (1.69)
Alda scale total score	4.32 (3.36)	3.97 (2.99)
**Non responders**	186	201
Age at interview	45.56 (11.82)	44.88 (12.66)
**Sex**		
Men	82 (44%)	84 (42%)
Women	105 (56%)	117 (58%)
Alda scale A score	1.63 (1.19)	1.73 (1.22)
Alda scale total B score	2.66 (1.64)	3.56 (1.80)
Alda scale total score	0.38 (0.76)	0.18 (0.49)
**Responders**	418	685
Age at interview	50.91(14.29)	47.50 (14.70)
**Sex**		
Men	170 (41%)	317 (46%)
Women	248 (59%)	368 (54%)
Alda scale A score	8.78 (1.05)	8.61 (1.08)
Alda scale total B score	1.66 (1.51)	2.50 (1.60)
Alda scale total score	7.12 (2.07)	6.11 (2.12)
**Continous (B < 4 exluded)**	772	1080
Age at interview	48.49 (13.73)	47.08 (14.05)
**Sex**		
Men	324 (42%)	471 (44%)
Women	448 (58%)	609 (56%)
Alda scale A score	6.25 (3.04)	6.66 (2.74)
Alda scale total B score	1.83 (1.29)	2.36 (1.16)
Alda scale total score	4.65 (3.31)	4.48 (2.89)

Data are n, n (%), or mean (SD). Alda scale refers to the Retrospective Criteria of Long-Term Treatment Response in Research Subjects with Bipolar Disorder; Non-resp = Li non responders (ALDA subscale A less than or equal to 3); Resp = Li responders (ALDA subscale A greater than or equal to 7).

For the association analyses of both GWAS1 and GWAS2, no HLA variant reached the Bonferroni significance (threshold of 5.2 × 10^−5^) in the additive model, neither for the continuous nor for the extreme phenotypes used to measure Li response with the Alda scale (comparison of extreme phenotypes, see “Materials and methods”). Nevertheless, several suggestive associations exhibiting a FDR < 0.25 were observed and are presented in Supplementary Table 2.

In the analysis of GWAS1 for the continuous trait (A score, individuals with B score > 4 were removed), the top ranked variants (*p*-values < 1 × 10^−3^ FDR = 0.12) were located within the *HLA-DRB1* and *HLA-DQB1* genes. For HLA-DRB1, Tyrosine/Leucine at position 37, Arginine at position 71, and Phenylalanine at position 67 were associated with a better response to Li (Table 2). Interestingly, these amino-acids notably belong to the HLA-DRB1 heavy chain encoding the HLA-DRB1*11:01 allele. Whereas weak response to Li was found to be associated with Leucine at position 26 of the *HLA-DQB1* locus (Table 2). None of these signals were replicated in the GWAS2. The amino-acid frequencies in the GWAS1 and GWAS2 were in agreement with those published in the reference cohorts from Allele frequency net database^25^ (Supplementary Table 3).

**Table 2:** Best results of association analysis of HLA with response to Li as continuous trait for group GWAS1 in additive model.

HLA protein, position, amino-acid(s)	Freq (%) GWAS1 (N = 772)	Beta GWAS1	*P*-value GWAS1	Q-value GWAS1
HLA-DRB1, 37, tyrosine and leucine	33.35	0.57	4.78 × 10^−4^	0.12
HLA-DRB1, 71, arginine	50.06	0.51	6.78 × 10^−4^	0.12
HLA-DQB1, 26, leucine	68.8	− 0.53	8.30 × 10^−4^	0.12
HLA-DRB1, 67, phenylalanine	16.65	0.68	9.80 × 10^−4^	0.12

Freq: frequency; Q-value: the *q*-value gives the expected positive false discovery rate.

In a second step, the meta-analysis of GWAS1 and GWAS2 (in the additive model) identified two signals almost reaching the 0.25-FDR threshold for suggestive associations (Table 3). Importantly, the associations were of similar magnitude and direction of effect for both the continuous and dichotomous traits. Once again, the two types of signals were related to the *HLA-DRB1* and *HLA-DQB1* loci and the frequency of their variants were also comparable to those of the reference cohorts from Allele frequency net database^25^ (Supplementary Table 3). The first signal in the *HLA-DQB1* locus which consisted of several amino-acids belonging to the HLA-DQB1*02 heavy chain was associated with no response to Li (Table 3). The second signal tags the amino-acid 74 of the HLA-DRB1 heavy chain. While Alanine or Leucine at position 74 are related to a better response to Li, Arginine or Glutamic acid at position 74 were associated to a weaker one (Table 3). For the analysis of the dichotomous phenotypes (defined by the Alda score ≥ 7 for the responders and ≤ 3 for non-responders), the frequency of the amino-acids Alanine or Leucine at position 74 was higher among the responders as compared to the non responders [72% vs 65% (GWAS1) and 73% vs 67% (GWAS2) in responders vs non-responder respectively]. Since the distribution of HLA alleles is known to exhibit South-North and/or East–West gradients even in European countries, we have specifically analysed the distribution of the alleles (A/L vs others) at position 74 in each country separately. For each country, we thus compared the allelic frequency under a dominant (patients carrying at least a A or a L allele) or a recessive (patients carrying only A or L alleles) model of the dichotomous phenotype of responders and non-responders (when N ≥ 15 in each group). Surprisingly, we observed that, in nine countries out of nine with sufficient number of patients in each dichotomous phenotype (Alda score ≥ 7 and Alda score ≤ 3), the frequency of patients carrying only A or L allele in the recessive model is systematically lower in non-responders than in responders (Table 4), suggesting a recessive effect. The probability of getting such a systematic difference in 9 countries out of 9 by chance is statistically very weak (*p* = 0.004). When comparing extreme groups (Alda score ≥ 9 and Alda score ≤ 3, see “Materials and methods”) in GWAS1 and GWAS2 for the allele A/L at position 74 in the recessive model, we obtained a FDR of 0.09 (Supplementary Tables 1 and 5), stronger than the one obtained for the additive model and confirming the recessive effect. Finally, it is worth mentioning that the HLA-DRB1*11:01 allele bears an Alanine 74 which confirms the above-mentioned favourable effect of such variant on responses to Li. Additionally, it is important to note that the HLA-DRB1 position 74 was previously reported to be in LD with the positions 37, 67, and 71 that came out strongly in GWAS1 (Table 2)^26^.

**Table 3:** Results of the meta-analysis for continuous and dichotomous response outcome in additive model.

HLA protein, position, amino-acid(s) or *HLA* allele	Meta-analysis continous scale			Meta-analysis dichotomous scale			AF GWAS1 (%)			AF GWAS2 (%)		
	Beta	*P*-value	Q	OR	*P*-value	Q	All (N = 772)	Non-responder (N = 186)	Responder (N = 418)	All (N = 1080)	Non-responder (N = 201)	Responder (N = 685)
HLA-DRB1, 74, alanine and leucine	0.32	1.94 × 10^−3^	0.27	1.32	2.22 × 10^−3^	0.27	70.27	65.24	72.37	71.19	67.41	73.07
HLA-DRB1, 74, arginine and glutamic acid	− 0.37	3.54 × 10^−3^	0.27	0.72	2.87 × 10^−3^	0.27	16.13	19.52	13.52	16.31	19.40	15.47
HLA-DQB1*02	− 0.33	3.96 × 10^−3^	0.27	0.75	5.40 × 10^−3^	0.27	21.70	26.20	20.33	20.57	22.89	18.61
HLA-DQB1, 74, alanine	− 0.33	3.87 × 10^−3^	0.27	0.75	5.40 × 10^−3^	0.27	21.70	26.20	20.33	20.57	22.89	18.61
HLA-DQB1, 71, lysine	− 0.33	3.87 × 10^−3^	0.27	0.75	5.40 × 10^−3^	0.27	21.70	26.20	20.33	20.57	22.89	18.61
HLA-DQB1, 55, leucine	− 0.33	3.87 × 10^−3^	0.27	0.75	5.40 × 10^−3^	0.27	21.70	26.20	20.33	20.57	22.89	18.61
HLA-DQB1, 52, leucine	− 0.33	3.87 × 10^−3^	0.27	0.75	5.40 × 10^−3^	0.27	21.70	26.20	20.33	20.57	22.89	18.61
HLA-DQB1, 47, phenylalanine	− 0.33	3.87 × 10^−3^	0.27	0.75	5.40 × 10^−3^	0.27	21.70	26.20	20.33	20.57	22.89	18.61
HLA-DQB1, 46, glutamic acid	− 0.33	3.87 × 10^−3^	0.27	0.75	5.40 × 10^−3^	0.27	21.70	26.20	20.33	20.57	22.89	18.61
HLA-DQB1, 37, isoleucine	− 0.33	3.87 × 10^−3^	0.27	0.75	5.40 × 10^−3^	0.27	21.70	26.20	20.33	20.57	22.89	18.61
HLA-DQB1, 30, serine	− 0.33	3.87 × 10^−3^	0.27	0.75	5.40 × 10^−3^	0.27	21.70	26.20	20.33	20.57	22.89	18.61
HLA-DQB1, 28, serine	− 0.33	3.87 × 10^−3^	0.27	0.75	5.40 × 10^−3^	0.27	21.70	26.20	20.33	20.57	22.89	18.61
HLA-DQB1, -10, serine	− 0.33	3.87 × 10^−3^	0.27	0.75	5.40 × 10^−3^	0.27	21.70	26.20	20.33	20.57	22.89	18.61

AF: allele frequency; Q-value: the *q*-value gives the expected positive false discovery rate; Non-resp = Li non responders (ALDA subscale A less than or equal to 3); Resp = Li responders (ALDA subscale A greater than or equal to 7).

**Table 4:** Country by country distribution of the patients carrying the HLA-DRB1 alleles A or L at position 74.

City/Country	N		A/L Allele frequency		Recessive model		Dominant model	
	Non- resp	Resp	Non-resp (%)	Resp (%)	Non-resp (%)	Resp (%)	Non-resp (%)	Resp (%)
**GWAS1**								
Cagliari/Italy	52	91	63.46	71.43	38.46	51.65	88.46	91.21
Halifax/Canada	55	150	58.18	71.00	32.73	52.00	83.64	90.00
San Diego/USA	79	58	68.99	75.00	46.84	62.07	91.14	87.93
Wuerzburg/Germany	25	30	62.00	76.67	36.00	56.67	88.00	96.67
**GWAS2**								
Barcelona/Spain	19	40	50.00	65.00	21.05	40.00	78.95	90.00
Canada	17	52	67.65	65.38	35.29	42.31	100.00	88.46
Paris/France	47	114	62.77	71.05	34.04	50.88	91.49	91.23
Romania	18	82	77.78	79.27	55.56	59.76	100.00	98.78
Sweden	39	200	71.79	77.25	51.28	59.50	92.31	95.00

Non-resp = Li non responders (ALDA subscale A less than or equal to 3); Resp = Li responders (ALDA subscale A greater than or equal to 7); N = number of patients in the group; recessive model = percentage of patients carrying only the A or L allele; dominant model = percentage of patients carrying at least one allele A or L.

In order to test dependence between the observed *HLA-DRB1* and *HLA-DQB1* signals, we evaluated the LD between their variants. We found a high level of correlation (r or D’ > 0.9) between HLA-DQB1*02 and two variants of *HLA-DRB1* (Supplementary Table 4). The r^2^ was found to be the higher (r^2^ > 0.5), between Alanine or Leucine at position 74 of HLA-DRB1 and HLA-DQB1*02 (Supplementary Table 4), which suggests a putative link between the *HLA-DRB1* and *HLA-DQB1*. We used Haploview^27^ to determine the haplotype between Alanine or Leucine at position 74 of HLA-DRB1 and HLA-DQB1*02 and we observed that all individuals carrying Alanine or Leucine at position 74 of HLA-DRB1 did not carry HLA-DQB1*02 and reciprocally. Given that Alanine and Leucine 74 have been found to be associated with good responders, while HLA-DQB1*02 with non-responders, it was not possible to determine whether the signal was due to the *HLA-DRB1* or to the *HLA-DQB1*, or both of them.

## Discussion

Understanding the mechanisms underlying inter-individual responses for a given therapeutic compound is a leading step towards precision medicine as, whatever the treatment efficacy, a subset of patients does not respond. Regarding BD, Li constitute the cornerstone of long-term therapy and management of the disease, but only around one-third of Li treated patients respond positively^28^. A great deal of research has thus been done to identify predictors of the degree of prevention against recurrences of mania and depression during long-term treatment. While several studies showed that Li response was polygenic, a large GWAS-based survey performed by the ConLiGen consortium in 2017, demonstrated that responses to Li were influenced both by the allostatic load of inter-individual SCZ polygenic scores and by genetic markers tagging the HLA antigen presentation pathway and genes encoding pro-inflammatory cytokines^19^.

Li has been demonstrated to exhibit potent anti-inflammatory properties mediated at least by the well-known inhibitory effects exerted on the Glycogen synthetase kinase-3-beta (GSK-3β) enzyme and downstream on the NF-κB pathway with consequent reduction of the pro-inflammatory cytokines TNF-α and IL-6 levels among others^29,30^. In addition, it has a potent anti-inflammatory action mediated both by the inhibition of IL-1β production and by the reduction of cyclooxygenase-2 expression while enhancing expression of IL-2 and IL-10 anti-inflammatory mediators. Finally, Li is also thought to modulate glial cellular inflammatory processes^31,32^. Given that the HLA system is central for the control of inflammatory processes, we imputed putative functional *HLA* variants within individuals from the above-mentioned GWAS and analyzed their potential association with response to Li.

Although no *HLA* variant per se reached the stringent Bonferroni significance threshold, we observed that several amino-acid positions had a low FDR score. Interestingly, all belonged to the HLA class-II cluster, suggesting the involvement of immune humoral processes in response to Li especially as some of them are located within the 3rd hypervariable (3HVR) sequences (amino-acids 67–74). Of interest, the 3HVR is the primary T-cell recognition site which conditions the immune adaptive processes mediated by the HLA class-II alleles^33^. Accordingly, the 3HVR is the core-associated region with immune processes including chronic inflammation and autoimmunity. It is thus important to mention that the *HLA* signal observed in the ConLiGen study^19^ tags the *HLA-DM* gene which encodes molecules pivotal for the HLA class-II antigen processing pathway by facilitating antigenic peptide loading to *HLA* class-II alleles^34^.

More precisely, in the analysis of patients belonging to GWAS1, we observed that within the HLA-DRB1 heavy chain, Tyrosine or Leucine at position 37, Arginine at position 71 and Phenylalanine at position 67, amino-acids all belonging to the HLA-DRB1*11:01 classical allele, are associated to a better response to Li. Such a finding could be discussed either at amino-acid or allele level. Indeed, Arginine 71 has previously been demonstrated to modulate disease severity in inflammatory/autoimmune settings. For example, in the context of inflammatory polyarthritis, a study evaluating the relationship between amino-acids at position 11, 71 and 74 and clinically defined inflammation showed that Arginine 71 belongs to haplotypes conferring protection against severe inflammation. In this study, while Valine at position 11 is the primary risk factor for severe clinical inflammation, a Serine at this position confers protection against clinically-defined inflammation and disease progression^35^. Of interest, HLA-DRB1*11:01 bears the reported protective haplotype against inflammation namely, Serine 11, Arginine 71 and Leucine 74. In addition, another study evaluating whether clinical severity in rheumatoid arthritis (RA) correlated with HLA genetic heterogeneity, demonstrated that among patients having the prototypic at risk shared epitope, consisting of specific amino-acids combinations at positions 70 to 74 of the 3HVR, Arginine 71 characterized patients who were non-producers of the rheumatoid factor autoantibody (RF), a subset of patients usually characterized with less aggressive and destructive disease as compared to those who produce RF^36^.

Our meta-analysis showed that patients having Alanine or Leucine at position 74 were more represented in the group of responders. Of note, HLA-DRB1*11:01 allele which bears Leucine at position 74 was also likely associated with good response. Patients with Arginine or Glutamic acid at position 74 belong to the non-responder subgroup. These associations were also observed in the analysis of extreme groups where homozygous subjects for the A/L alleles were always found in smaller numbers in the extreme non-responders as compared to responders (Table 4, Supplementary Table 1), altogether suggesting a recessive effect. The beneficial effect exerted by the HLA-DRB1*11:01 allele can be discussed regarding its involvement in immune disease settings. A Brazilian study of the genetic influence on multiple sclerosis, a chronic autoimmune condition, showed that beside the canonical at risk HLA-DRB1*15 allele, the HLA-DRB1*11 variant exerts a strong protective effect against the disorder^37^. Such protective effect against multiple sclerosis was also reported in ethnically distant population-groups^38^. Moreover, a recent study allowed us to establish a link between our finding and the signal identified in the primary ConLiGen GWAS study concerning the association with the HLA-DM region^19^. Indeed, the authors, analyzing the mechanism underlying the protective status conferred by HLA-DRB1*01:01 and HLA-DRB1*11:01 alleles against multiple sclerosis, demonstrated the involvement of the HLA-DM-mediated lower efficiency to bind antigenic peptide to the antigen peptide groove of the protective allele with consequent failure of peptide presentation at the cell surface and possible alleviation of inflammatory/autoimmune processes^39^. In another immune condition, namely chronic Hepatitis C Virus infection, the HLA-DRB1*11 was demonstrated to mediate protection against liver damage^40^. Altogether, these data suggest that good Li responders are associated with HLA variants conveying low inflammatory characteristics.

Concerning the non-responder patient group, an association was found with Glutamic acid or Arginine at position 74 in *HLA-DRB1*. The latter has been shown to drive the risk of developing Grave’s disease, a common inflammatory/auto-immune disorder either in patients bearing the usually associated HLA-DRB1*03 specificity or in affected non-DR3 Graves patients^41^. In addition, we also observed an association between weak response to Li and multiple amino-acids scattered along the heavy chain of the *HLA-DQB1* gene, almost all corresponding to the HLA-DQB1*02 allele. This association may suggest the involvement of the well-known *HLA-8.1* ancestral haplotype (A*01 ~ B*08 ~ DRB1*03 ~ DQB1*02), which is characterized by a steady state pro-inflammatory status in healthy subjects and is the most associated *HLA* haplotype with inflammatory/autoimmune disorders. Another possibility explaining the association with HLA-DQB1*02 could be merely a LD with another *HLA* variant, not uncovered in the present study such as HLA-DRB1*03 known to be in LD with HLA-DQB1*02 and to bear the non-response associated Arginine 74. This hypothesis needs to be confirmed in larger cohorts of patients as it may have practical therapeutic implications such as add-on treatments of anti-inflammatory drugs in non-responders to Li.

Our study was performed on patients coming from multiple countries, which inevitably introduce heterogeneity in terms of data collection, methods of diagnosis/evaluation of the phenotype and information on patients such as comorbidities, overall constitutes possible confounding effects. Within this line, the fact that the distribution of Alda scores in the GWAS1 group was slightly more variable than that observed in the GWAS2 group and that patients belonging to the GWAS1 were slightly older than that of the GWAS2 group (Table 1), may also contribute to differences in the analysis of the two groups. In spite of these limits, the association between the HLA-DRB1 position 74 A/L allele and responders was consistently observed under the recessive model with extreme phenotypes in all the countries tested in the GWAS1 and in the GWAS2. We have already mentioned that the recessive effect for HLA-DRB1 cannot be dissociated from a dominant effect for the HLA-DQB1*02 allele in terms of genetic association. Such recessive-based observation may reflect the characteristics of the HLA system (and other innate immune sensors) in terms of structure, function and evolution where the heterozygosity advantage was selected to better fight pathogen diversity, a recessive status may prevent overwhelmed inflammatory processes^42–44^.

Altogether, beside the well-documented anti-inflammatory effect exerted by Li on the innate GSK-3β pathways and consequently on circulating pro- and anti-inflammatory cytokines, our observations indicate that response to Li in BD patients may be underpinned by adaptive HLA-mediated inflammatory processes, fitting with the known anti-inflammatory effect driven by Li that may contribute to its therapeutic efficiency^31^. Our genetic findings showing an association between HLA-mediated lower inflammation in patients with efficient response to Li, may suggest that non-responders among BD patients could have inflammatory backgrounds that override Li mediated anti-inflammatory properties.

## Materials and methods

### Study population

This study includes GWAS genotype and phenotype data of Li-treated BD patients from 22 participating sites belonging to the International Consortium on Lithium Genetics (ConLiGen)^16^. The whole cohort consisted of 2588 patients resulting from two GWAS of 1187 and 1401 individuals named GWAS1 and GWAS2 in the original publication^16^. Samples were collected and genotyped in two distinct steps, determining the GWAS1 and GWAS2 groups [detailed rationale is available in the appendix of Hou et al*.*^16^]. The various cohorts were initially developed separately and various chips were used for genotyping^16^. We kept the same distribution of the samples and the names GWAS1 and GWAS2. After removal of Japanese, Taiwanese and Sardinian patients given the poor applicability of *HLA* imputation in these population-groups on the one hand, and the difference in HLA frequencies among the Sardinian population on the other, 862 and 1309 patients belonging respectively to the GWAS1 and GWAS2 were subjected to analysis. We performed a quality control of the genotypes for each patient. As cryptic relatedness between individuals can introduce a bias in association analysis and in the following meta-analysis, we computed identity-by-descent (IBD) between all pairs of individuals for the two groups and removed one individual of the related pair if a second degree related can be detected (N = 14 for GWAS1 and N = 11 for the GWAS2). We also removed one patient in the GWAS1 and six patients in the GWAS2 because of an excess of heterozygosity, suggesting a poor DNA quality. After these two steps, 847 patients from GWAS1 and 1292 from GWAS2 remained for analysis.

The Conligen consortium used data obtained through an international collaboration. The University of Heidelberg Ethics Committee provided the central ethics approval for the consortium. Written informed consent was obtained from each patient (they were all aged > 18) according to the study protocols of the participating cohorts and their respective institutions. All procedures were performed in accordance with the guidelines of the institutional research committees and of the Helsinki Declaration.

### Study cohort phenotypes

From the discovery GWAS participants^16^, long-term treatment responses to Li were evaluated using the Alda scale. Briefly, the Alda scale used criterion A to rate the degree of response (activity of illness while on adequate Li treatment) on a 10-point scale and criterion B which establishes the relationship between improvement and treatment (i.e. number of episodes off treatment, frequency of episode off treatment, duration of treatment, compliance, use of additional treatment). The total score is obtained by subtracting score B from score A and negative scores are set to 0, so that the total score ranges from 0 to 10.

In the present study, we performed 3 types of phenotype analyses in order to dissect the genetic associations with Li response:

(1) A continuous phenotype (range 0–10) using the A score, excluding all individuals with a total B score greater than 4 for a dimensional assessment of Li response. In this study, there were 772 patients studied in GWAS1 and 1080 patients studied in GWAS2 by linear regression with the continuous phenotype in the additive model.

(2) A dichotomous phenotype comparing good vs non responders to Li, defined by an A Alda score ≥ 7 for responders and ≤ 3 for non-responders. In this comparative study (in the additive model), there were 186 non-responders compared with 418 responders from GWAS1, and 201 non-responders compared with 685 responders from GWAS2. To take into account the variations of distribution of HLA alleles according to countries, we also checked country by country the frequency of patients carrying the HLA-DRB1 A or L alleles at position 74 in the non-responder and responder groups, when these groups had enough patients (N ≥ 15) to compute a reliable frequency. We added the Sardinian population (comprising 52 non-responders compared with 91 good responders, Table 4), even if it was excluded from the statistical analysis.

(3) We also performed a country-by-country analysis in extreme phenotypes defined by an A Alda score ≥ 9 for responders and ≤ 3 for non-responders: there were 186 non-responders compared with 275 responders from GWAS1, and 201 non-responders compared with 362 responders from GWAS2. In Sardinian population, there were 52 non-responders compared with 39 good responders (Supplementary Table 1). We also computed a FDR value comparing the non-responders with responders in the recessive model with this extreme phenotype (Supplementary Table 5).

### Imputation of HLA variants

The SNP2HLA software^45^ has been developed to impute directly *HLA* class-I and class II-classical alleles at the 4-digit level from the chromosome 6 genotypic data of each patient. In addition, the software computes directly the amino-acid variants from the imputed 4-digit HLA alleles, allowing to test associations also at the level of the HLA protein amino-acid variants (including the ones constituting the peptide antigen presenting groove) thus facilitating the biological interpretation. A total of 1708 imputed variants (433 classical *HLA* alleles and 1275 amino-acid variations) were generated. The variants with minor allele frequency < 0.05 and/or *p*-value for Hardy–Weinberg departure test < 1 × 10^−6^ were removed. The resulting 85 classical *HLA* alleles and 880 amino-acid variations were subjected to the statistical analysis pipeline. To validate the accuracy of the imputation, we compared the frequency of the variants with those listed in the Allele frequency net database^25^. Given that the studied populations were from European descent, we choose available representative populations from France (France, Rennes, N = 200; France, west, N = 100) and from Germany (Germany, Essen, N = 174) in the Allele Frequency Net Database with alleles given in at least four digits. The best variants obtained from our association analysis had a rather strong MAF, and since the MAF found in the control populations were similar it was sufficient to confirm the quality of the HLA imputation for thee variants, even if these control populations had a relatively modest size. For the amino-acid frequencies we used the tool of translation from Allele frequency net database^25^.

### Statistical analysis

For the 965 HLA imputed variants having passed the quality controls, associations between HLA amino-acids or alleles and response to Li were tested. The statistical analysis was performed by a multivariate linear or logistic regression using PLINK software^46^ under additive model for response to Li (continuous or binary phenotypes). To correct for possible population stratification, we performed principal component analysis (PCA) on the genotypes and used the top two principal components as covariates in statistical analyses. To avoid spurious association, we added age and gender of patients as covariates. Finally, a classical meta-analysis was performed between associations resulting from GWAS1 and GWAS2 for two phenotypes (continuous (1) and dichotomous (2)) using PLINK. We used the Bonferroni threshold (for 965 tests − 85 classical *HLA* alleles + 880 amino-acid-, *p*-value < 5.2 × 10^−5^) to determine the significance of the associations and we also took the false discovery rate (FDR) < 0.25 for the suggestive associations.

## Supplementary Information

Supplementary Information.
