# Supplementary material for: HLA-DRB1 and HLA-DQB1 genetic diversity modulates response to lithium in bipolar affective disorders
Source: Sci Rep. 2021 Sep 8;11:17823. doi: 10.1038/s41598-021-97140-7 (PMC8426488; doi:10.1038/s41598-021-97140-7)
Supplement: Supplementary file 1 — Supplementary Information. [file 41598_2021_97140_MOESM1_ESM.docx]

**SUPPLEMENTARY DOCUMENT:**

**HLA-DRB1 and HLA-DQB1 genetic diversity modulates response to Lithium in Bipolar Affective Disorders**

Sigrid Le Clerc, Laura Lombardi, Bernhard T. Baune, Azmeraw T. Amare, Klaus Oliver Schubert, Liping Hou, Scott R Clark, Sergi Papiol, Micah Cearns, Urs Heilbronner, Franziska Degenhardt, Fasil Tekola-Ayele, Yi-Hsiang Hsu, Tatyana Shekhtman, Mazda Adli, Nirmala Akula, Kazufumi Akiyama, Raffaella Ardau, Bárbara Arias, Jean-Michel Aubry, Lena Backlund, Abesh Kumar Bhattacharjee, Frank Bellivier, Antonio Benabarre, Susanne Bengesser, Joanna M Biernacka, Armin Birner, Clara Brichant-Petitjean, Pablo Cervantes, Hsi-Chung Chen, Caterina Chillotti, Sven Cichon, Cristiana Cruceanu, Piotr M Czerski, Nina Dalkner, Alexandre Dayer, Maria Del Zompo, J Raymond DePaulo, Bruno Étain, Stephane Jamain, Peter Falkai, Andreas J Forstner, Louise Frisen, Mark A Frye, Janice M Fullerton, Sébastien Gard, Julie S Garnham, Fernando S Goes, Maria Grigoroiu-Serbanescu, Paul Grof, Ryota Hashimoto, Joanna Hauser, Stefan Herms, Per Hoffmann, Esther Jiménez, Jean-Pierre Kahn, Layla Kassem, Po-Hsiu Kuo, Tadafumi Kato, John R Kelsoe, Sarah Kittel-Schneider, Ewa Ferensztajn-Rochowiak, Barbara König, Ichiro Kusumi, Gonzalo Laje, Mikael Landén, Catharina Lavebratt, Susan G Leckband, Alfonso Tortorella, Mirko Manchia, Lina Martinsson, Michael J McCarthy, Susan L McElroy, Francesc Colom, Vincent Millischer, Marina Mitjans, Francis M Mondimore, Palmiero Monteleone, Caroline M Nievergelt, Markus M Nöthen, Tomas Novák, Claire O'Donovan, Norio Ozaki, Urban Ösby, Andrea Pfennig, James B Potash, Andreas Reif, Eva Reininghaus, Guy A Rouleau, Janusz K Rybakowski, Martin Schalling, Peter R Schofield, Barbara W Schweizer, Giovanni Severino, Paul D Shilling**,** Katzutaka Shimoda, Christian Simhandl, Claire M Slaney, Claudia Pisanu, Alessio Squassina, Thomas Stamm, Pavla Stopkova, Mario Maj, Gustavo Turecki, Eduard Vieta, Julia Veeh, Stephanie H Witt, Adam Wright, Peter P Zandi, Philip B Mitchell, Michael Bauer, Martin Alda, Marcella Rietschel, Francis J McMahon, Thomas G Schulze**,** Jean-Louis Spadoni, Wahid Boukouaci, Jean-Romain Richard, Philippe Le Corvoisier, Caroline Barrau, Jean-François Zagury, Marion Leboyer, Ryad Tamouza

**Supplementary Tables**

**Supplementary table 1: Country by country distribution of the patients carrying the HLA-DRB1 alleles A or L at position 74 (extreme phenotype groups Alda ≥ 9 vs Alda ≤ 3)**

|  | City/Country | N | | A/L Allele frequency | | Recessive model | | Dominant model | |
| --- | --- | --- | --- | --- | --- | --- | --- | --- | --- |
|  |  | Non- resp | Resp | Non-resp (%) | Resp (%) | Non-resp (%) | Resp (%) | Non-resp (%) | Resp (%) |
| GWAS1 | Cagliari/Italy | 52 | 39 | 63.46 | 66.67 | 38.46 | 46.15 | 88.46 | 87.18 |
|  | Halifax/Canada | 55 | 132 | 58.18 | 73.48 | 32.73 | 55.30 | 83.64 | 91.67 |
|  | San Diego/USA | 79 | 20 | 68.99 | 62.50 | 46.84 | 55.00 | 91.14 | 70.00 |
|  | Wuerzburg/Germany | 25 | 15 | 62.00 | 83.33 | 36.00 | 66.67 | 88.00 | 100.00 |
| GWAS2 | Barcelona/Spain | 19 | 16 | 50.00 | 56.25 | 21.05 | 31.25 | 78.95 | 81.25 |
|  | Canada | 17 | 17 | 67.65 | 58.82 | 35.29 | 41.18 | 100.00 | 76.47 |
|  | Paris/France | 47 | 68 | 62.77 | 75.74 | 34.04 | 58.82 | 91.49 | 92.65 |
|  | Romania | 18 | 50 | 77.78 | 81.00 | 55.56 | 62.00 | 100.00 | 100.00 |
|  | Sweden | 39 | 131 | 71.79 | 78.63 | 51.28 | 62.60 | 92.31 | 94.66 |

Non-resp = Li non responders (ALDA subscale A less than or equal to 3); Resp = Li responders (ALDA subscale A greater than or equal to 9); N = number of patients in the group; recessive model = percentage of patients carrying only the A or L allele; dominant model = percentage of patients carrying at least one allele A or L.

**Supplementary table 2: Results of association analysis of HLA with response to Li as continuous trait for group GWAS1 with FDR < 0.25 (additive model)**

| **SNP** | **BETA** | **SE** | **P** | **Q** | **info_imputation** |
| --- | --- | --- | --- | --- | --- |
| **AA_DRB1_37_32660037_YL** | **0.57** | **0.16** | **4.78E-04** | **0.12** | **0.97** |
| **AA_DRB1_71_32659935_R** | **-0.51** | **0.15** | **6.78E-04** | **0.12** | **0.93** |
| **AA_DQB1_26_32740759_L** | **0.53** | **0.16** | **8.30E-04** | **0.12** | **0.98** |
| **AA_DRB1_67_32659947_F** | **0.69** | **0.21** | **9.79E-04** | **0.12** | **0.91** |
| **AA_DQB1_182_32737742_S** | **0.51** | **0.16** | **1.50E-03** | **0.12** | **0.98** |
| **AA_DQB1_140_32737868_A** | **0.51** | **0.16** | **1.50E-03** | **0.12** | **0.94** |
| **AA_DQB1_182_32737742_N** | **0.50** | **0.16** | **1.68E-03** | **0.12** | **0.99** |
| **AA_DQB1_140_32737868_T** | **0.50** | **0.16** | **1.68E-03** | **0.12** | **0.94** |
| **AA_DRB1_37_32660037_Y** | **0.52** | **0.17** | **1.75E-03** | **0.12** | **0.96** |
| **HLA_DQB1_0602** | **-0.72** | **0.23** | **1.86E-03** | **0.12** | **0.95** |
| **AA_DRB1_71_32659935_KA** | **-0.50** | **0.16** | **2.09E-03** | **0.12** | **0.96** |
| **HLA_DRB1_1501** | **-0.70** | **0.23** | **2.17E-03** | **0.12** | **0.98** |
| AA_DQB1_-5_32742280_L | -0.56 | 0.19 | 2.88E-03 | 0.13 | 0.98 |
| AA_DRB1_231_32656010_Q | 0.98 | 0.33 | 3.35E-03 | 0.13 | 0.91 |
| AA_DRB1_71_32659935_A | -0.65 | 0.22 | 3.74E-03 | 0.13 | 0.99 |
| HLA_DRB1_15 | -0.65 | 0.22 | 3.74E-03 | 0.13 | 0.99 |
| AA_DRB1_-1_32665412_S | -0.65 | 0.22 | 3.74E-03 | 0.13 | 0.99 |
| AA_DRB1_16_32660100_H | 0.97 | 0.33 | 4.00E-03 | 0.13 | 0.87 |
| AA_DRB1_16_32660100_Y | 0.97 | 0.33 | 4.00E-03 | 0.13 | 0.87 |
| AA_DRB1_13_32660109_G | 0.97 | 0.33 | 4.00E-03 | 0.13 | 0.87 |
| AA_DQB1_-5_32742280_PS | -0.50 | 0.17 | 4.11E-03 | 0.13 | 0.98 |
| HLA_DPB1_0201 | 0.60 | 0.21 | 5.36E-03 | 0.15 | 0.90 |
| AA_DQB1_125_32737913_G | -0.49 | 0.17 | 5.36E-03 | 0.15 | 0.98 |
| HLA_DQB1_06 | -0.49 | 0.17 | 5.36E-03 | 0.15 | 0.98 |
| AA_DQB1_125_32737913_AS | -0.48 | 0.17 | 6.12E-03 | 0.15 | 0.98 |
| AA_B_156_31432003_LW | -0.44 | 0.16 | 6.13E-03 | 0.15 | 0.96 |
| AA_B_156_31432003_RD | -0.44 | 0.16 | 6.13E-03 | 0.15 | 0.96 |
| AA_DRB1_13_32660109_FG | 0.53 | 0.19 | 6.40E-03 | 0.15 | 0.96 |
| AA_DRB1_71_32659935_KR | -0.45 | 0.17 | 6.54E-03 | 0.15 | 0.95 |
| AA_A_156_30019219_RL | 0.47 | 0.17 | 6.61E-03 | 0.15 | 0.97 |
| AA_A_156_30019219_Q | 0.56 | 0.21 | 8.54E-03 | 0.16 | 0.98 |
| AA_DQB1_87_32740576_F | -0.50 | 0.19 | 8.82E-03 | 0.16 | 0.97 |
| AA_B_325_31430282_C | -0.41 | 0.16 | 8.89E-03 | 0.16 | 0.98 |
| HLA_DPB1_02 | 0.56 | 0.21 | 9.09E-03 | 0.16 | 0.91 |
| AA_DRB1_86_32659890 | -0.40 | 0.15 | 9.41E-03 | 0.16 | 0.92 |
| AA_DRB1_-1_32665412_x | 0.91 | 0.35 | 9.58E-03 | 0.16 | 0.88 |
| AA_DRB1_-16_32665457_x | 0.91 | 0.35 | 9.58E-03 | 0.16 | 0.88 |
| AA_DRB1_-17_32665460_x | 0.91 | 0.35 | 9.58E-03 | 0.16 | 0.88 |
| AA_DRB1_-24_32665481_x | 0.91 | 0.35 | 9.58E-03 | 0.16 | 0.88 |
| AA_DRB1_-25_32665484_x | 0.91 | 0.35 | 9.58E-03 | 0.16 | 0.88 |
| AA_DQB1_87_32740576_LY | -0.49 | 0.19 | 1.01E-02 | 0.16 | 0.97 |
| AA_DRB1_189_32656611_R | 0.88 | 0.34 | 1.03E-02 | 0.16 | 0.92 |
| AA_DRB1_13_32660109_SRY | 0.41 | 0.16 | 1.04E-02 | 0.16 | 0.96 |
| AA_DRB1_37_32660037_NS | 0.39 | 0.15 | 1.09E-02 | 0.16 | 0.98 |
| AA_B_97_31432180_SWV | -0.42 | 0.16 | 1.10E-02 | 0.16 | 0.98 |
| AA_B_325_31430282_S | -0.40 | 0.16 | 1.11E-02 | 0.16 | 0.97 |
| AA_DRB1_74_32659926_RE | -0.53 | 0.21 | 1.15E-02 | 0.16 | 0.93 |
| AA_DRB1_37_32660037_NF | -0.38 | 0.15 | 1.27E-02 | 0.17 | 0.97 |
| HLA_DQB1_03 | 0.41 | 0.16 | 1.29E-02 | 0.17 | 0.94 |
| AA_DQB1_55_32740672_P | 0.41 | 0.16 | 1.29E-02 | 0.17 | 0.94 |
| AA_DRB1_70_32659938_D | 0.38 | 0.15 | 1.34E-02 | 0.17 | 0.96 |
| AA_B_97_31432180_RT | -0.39 | 0.16 | 1.39E-02 | 0.18 | 0.98 |
| AA_B_97_31432180_SW | -0.41 | 0.17 | 1.49E-02 | 0.18 | 0.97 |
| AA_B_95_31432186_W | 0.51 | 0.21 | 1.57E-02 | 0.19 | 0.95 |
| AA_B_282_31430958_V | 0.38 | 0.16 | 1.70E-02 | 0.20 | 0.97 |
| AA_B_156_31432003_L | -0.37 | 0.16 | 1.77E-02 | 0.20 | 0.96 |
| AA_B_156_31432003_Lx | -0.37 | 0.16 | 1.77E-02 | 0.20 | 0.96 |
| AA_B_282_31430958_I | 0.37 | 0.16 | 1.83E-02 | 0.21 | 0.96 |
| AA_B_97_31432180_SWN | -0.38 | 0.16 | 1.90E-02 | 0.21 | 0.97 |
| AA_B_97_31432180_SV | -0.39 | 0.17 | 1.95E-02 | 0.21 | 0.98 |
| AA_DPB1_205_33161588_V | -0.57 | 0.24 | 2.02E-02 | 0.22 | 0.80 |
| AA_B_97_31432180_S | -0.40 | 0.17 | 2.20E-02 | 0.23 | 0.97 |
| AA_DRB1_70_32659938_Q | -0.35 | 0.15 | 2.22E-02 | 0.23 | 0.96 |
| AA_A_95_30019036_L | 0.53 | 0.23 | 2.30E-02 | 0.23 | 0.98 |
| AA_B_305_31430889_A | 0.36 | 0.16 | 2.36E-02 | 0.23 | 0.98 |
| AA_B_97_31432180_SNV | -0.37 | 0.16 | 2.41E-02 | 0.23 | 0.97 |
| AA_B_305_31430889_T | 0.35 | 0.16 | 2.52E-02 | 0.23 | 0.97 |
| AA_DRB1_74_32659926_AL | -0.37 | 0.16 | 2.59E-02 | 0.23 | 0.95 |
| HLA_C_12 | 0.72 | 0.32 | 2.65E-02 | 0.23 | 0.98 |
| AA_DRB1_57_32659977_S | 0.75 | 0.34 | 2.70E-02 | 0.23 | 0.88 |
| AA_DRB1_47_32660007 | -0.33 | 0.15 | 2.90E-02 | 0.23 | 0.98 |
| AA_DQB1_185_32737733_T | 0.46 | 0.21 | 2.93E-02 | 0.23 | 0.98 |
| AA_DRB1_37_32660037_YF | 0.33 | 0.15 | 3.13E-02 | 0.23 | 0.97 |
| AA_DQB1_74_32740615_S | 0.42 | 0.20 | 3.17E-02 | 0.23 | 0.94 |
| AA_DQB1_71_32740624_KT | 0.42 | 0.20 | 3.17E-02 | 0.23 | 0.95 |
| AA_DQB1_26_32740759_G | 0.42 | 0.20 | 3.17E-02 | 0.23 | 0.94 |
| AA_B_97_31432180_SN | -0.35 | 0.16 | 3.24E-02 | 0.23 | 0.97 |
| AA_DPB1_194_33161555_x | -0.60 | 0.28 | 3.28E-02 | 0.23 | 0.72 |
| AA_DPB1_205_33161588_x | -0.60 | 0.28 | 3.28E-02 | 0.23 | 0.72 |
| AA_DPB1_215_33161618_x | -0.60 | 0.28 | 3.28E-02 | 0.23 | 0.72 |
| AA_A_76_30018738_A | -0.40 | 0.19 | 3.30E-02 | 0.23 | 0.97 |
| AA_DQB1_185_32737733_I | 0.45 | 0.21 | 3.32E-02 | 0.23 | 0.98 |
| AA_DRB1_26_32660070_Y | -0.50 | 0.23 | 3.38E-02 | 0.23 | 0.98 |
| AA_DRB1_37_32660037_SY | -0.33 | 0.15 | 3.39E-02 | 0.23 | 0.97 |
| AA_DRB1_13_32660109_SR | 0.32 | 0.15 | 3.52E-02 | 0.23 | 0.97 |
| HLA_A_24 | 0.56 | 0.27 | 3.55E-02 | 0.23 | 0.99 |
| HLA_DQB1_0201 | -0.50 | 0.24 | 3.65E-02 | 0.23 | 0.97 |
| HLA_C_07 | -0.34 | 0.16 | 3.72E-02 | 0.23 | 0.99 |
| AA_C_194_31346208_L | -0.34 | 0.16 | 3.72E-02 | 0.23 | 0.99 |
| AA_C_184_31346238_P | -0.34 | 0.16 | 3.72E-02 | 0.23 | 0.99 |
| AA_C_184_31346238_Px | -0.34 | 0.16 | 3.72E-02 | 0.23 | 0.99 |
| AA_C_-9_31347781_G | -0.34 | 0.16 | 3.72E-02 | 0.23 | 0.99 |
| AA_DRB1_37_32660037_N | -0.38 | 0.18 | 3.77E-02 | 0.23 | 0.98 |
| AA_DQA1_207_32718440_M | -0.38 | 0.19 | 4.00E-02 | 0.23 | 0.99 |
| AA_DQA1_207_32718440_V | -0.38 | 0.19 | 4.00E-02 | 0.23 | 0.99 |
| HLA_A_2402 | 0.55 | 0.27 | 4.18E-02 | 0.23 | 0.95 |
| AA_B_-21_31432904_M | -0.33 | 0.16 | 4.33E-02 | 0.23 | 0.98 |
| AA_B_-23_31432910_L | -0.33 | 0.16 | 4.33E-02 | 0.23 | 0.98 |
| AA_DRB1_13_32660109_HG | 0.39 | 0.19 | 4.39E-02 | 0.23 | 0.94 |
| AA_DRB1_77_32659917 | -0.48 | 0.24 | 4.40E-02 | 0.23 | 0.98 |
| AA_DRB1_74_32659926_R | -0.48 | 0.24 | 4.40E-02 | 0.23 | 0.98 |
| HLA_DRB1_03 | -0.48 | 0.24 | 4.40E-02 | 0.23 | 0.99 |
| HLA_DRB1_0301 | -0.48 | 0.24 | 4.40E-02 | 0.23 | 0.99 |
| AA_DQB1_167_32737787_R | 0.39 | 0.19 | 4.45E-02 | 0.23 | 0.98 |
| AA_C_339_31345102_A | -0.32 | 0.16 | 4.47E-02 | 0.23 | 0.99 |
| AA_C_339_31345102_T | -0.32 | 0.16 | 4.47E-02 | 0.23 | 0.99 |
| AA_C_326_31345141_C | -0.32 | 0.16 | 4.47E-02 | 0.23 | 0.99 |
| AA_C_326_31345141_S | -0.32 | 0.16 | 4.47E-02 | 0.23 | 0.99 |
| AA_C_307_31345745_M | -0.32 | 0.16 | 4.47E-02 | 0.23 | 0.99 |
| AA_C_307_31345745_V | -0.32 | 0.16 | 4.47E-02 | 0.23 | 0.99 |
| AA_C_305_31345751_A | -0.32 | 0.16 | 4.47E-02 | 0.23 | 0.99 |
| AA_C_305_31345751_T | -0.32 | 0.16 | 4.47E-02 | 0.23 | 0.99 |
| AA_C_295_31345781_A | -0.32 | 0.16 | 4.47E-02 | 0.23 | 0.99 |
| AA_C_295_31345781_V | -0.32 | 0.16 | 4.47E-02 | 0.23 | 0.99 |
| AA_C_285_31345811_M | -0.32 | 0.16 | 4.47E-02 | 0.23 | 0.99 |
| AA_C_285_31345811_Mx | -0.32 | 0.16 | 4.47E-02 | 0.23 | 0.99 |
| AA_C_273_31345971_R | -0.32 | 0.16 | 4.47E-02 | 0.23 | 0.99 |
| AA_C_273_31345971_S | -0.32 | 0.16 | 4.47E-02 | 0.23 | 0.99 |
| AA_C_261_31346007_M | -0.32 | 0.16 | 4.47E-02 | 0.23 | 0.99 |
| AA_C_261_31346007_V | -0.32 | 0.16 | 4.47E-02 | 0.23 | 0.99 |
| AA_C_194_31346208_V | -0.32 | 0.16 | 4.47E-02 | 0.23 | 0.99 |
| AA_C_-9_31347781_A | -0.32 | 0.16 | 4.47E-02 | 0.23 | 0.99 |
| HLA_B_08 | -0.49 | 0.24 | 4.48E-02 | 0.23 | 0.99 |
| HLA_B_0801 | -0.49 | 0.24 | 4.48E-02 | 0.23 | 0.99 |
| AA_C_9_31347600_D | -0.31 | 0.15 | 4.61E-02 | 0.23 | 0.99 |
| AA_B_-21_31432904_T | -0.32 | 0.16 | 4.66E-02 | 0.23 | 0.97 |
| AA_B_-23_31432910_R | -0.32 | 0.16 | 4.66E-02 | 0.23 | 0.97 |
| AA_DRB1_67_32659947_I | -0.31 | 0.16 | 4.82E-02 | 0.23 | 0.94 |
| AA_A_62_30018696_RE | 0.36 | 0.18 | 4.84E-02 | 0.23 | 0.98 |
| AA_DQB1_167_32737787_H | 0.38 | 0.19 | 4.86E-02 | 0.23 | 0.97 |
| AA_DQB1_26_32740759_Y | 0.38 | 0.19 | 4.86E-02 | 0.23 | 0.92 |
| AA_DQB1_13_32740798_A | 0.38 | 0.19 | 4.86E-02 | 0.23 | 0.92 |
| AA_DQB1_13_32740798_G | 0.38 | 0.19 | 4.86E-02 | 0.23 | 0.93 |
| AA_A_9_30018537_FT | 0.33 | 0.17 | 4.99E-02 | 0.23 | 0.99 |
| HLA_DQA1_0102 | -0.36 | 0.19 | 5.04E-02 | 0.23 | 0.99 |
| AA_A_62_30018696_E | 0.47 | 0.24 | 5.24E-02 | 0.23 | 0.99 |
| AA_C_152_31346921_A | -0.31 | 0.16 | 5.29E-02 | 0.23 | 0.99 |
| AA_DPB1_11_33156445 | -0.35 | 0.18 | 5.35E-02 | 0.23 | 0.98 |
| AA_DPB1_215_33161618_I | -0.52 | 0.27 | 5.38E-02 | 0.23 | 0.73 |
| AA_DQB1_-10_32742295_A | -0.34 | 0.18 | 5.43E-02 | 0.23 | 0.98 |
| AA_A_44_30018642 | -0.41 | 0.21 | 5.43E-02 | 0.23 | 0.99 |
| AA_A_67_30018711 | -0.41 | 0.21 | 5.43E-02 | 0.23 | 0.99 |
| AA_A_150_30019201 | -0.41 | 0.21 | 5.43E-02 | 0.23 | 0.99 |
| AA_A_156_30019219_R | -0.41 | 0.21 | 5.43E-02 | 0.23 | 0.99 |
| AA_A_158_30019225 | -0.41 | 0.21 | 5.43E-02 | 0.23 | 0.99 |
| HLA_A_01 | -0.41 | 0.21 | 5.43E-02 | 0.23 | 0.99 |
| HLA_A_0101 | -0.41 | 0.21 | 5.43E-02 | 0.23 | 0.99 |
| AA_DQB1_9_32740810_F | -0.41 | 0.21 | 5.52E-02 | 0.23 | 0.96 |
| AA_DQB1_9_32740810_YL | -0.41 | 0.21 | 5.52E-02 | 0.23 | 0.96 |
| AA_DRB1_11_32660115_SVL | -0.33 | 0.17 | 5.53E-02 | 0.23 | 0.99 |
| AA_C_24_31347555 | -0.29 | 0.15 | 5.60E-02 | 0.23 | 0.99 |
| AA_C_9_31347600_DF | -0.29 | 0.15 | 5.60E-02 | 0.23 | 0.99 |
| AA_DRB1_11_32660115_PD | -0.40 | 0.21 | 5.61E-02 | 0.23 | 0.99 |
| AA_DRB1_13_32660109_SRH | 0.30 | 0.16 | 5.67E-02 | 0.23 | 0.96 |
| AA_B_156_31432003_D | -0.35 | 0.18 | 5.91E-02 | 0.23 | 0.98 |
| AA_B_156_31432003_Dx | -0.35 | 0.18 | 5.91E-02 | 0.23 | 0.98 |
| AA_B_9_31432689_D | -0.46 | 0.24 | 5.92E-02 | 0.23 | 0.98 |
| AA_B_74_31432494 | -0.30 | 0.16 | 6.08E-02 | 0.23 | 0.97 |
| AA_DRB1_142_32657452_M | -0.40 | 0.21 | 6.10E-02 | 0.23 | 0.99 |
| AA_DRB1_142_32657452_V | -0.40 | 0.21 | 6.10E-02 | 0.23 | 0.98 |
| AA_DRB1_133_32657479_L | -0.40 | 0.21 | 6.10E-02 | 0.23 | 0.99 |
| AA_DRB1_133_32657479_R | -0.40 | 0.21 | 6.10E-02 | 0.23 | 0.98 |
| AA_DQB1_74_32740615_A | -0.34 | 0.18 | 6.18E-02 | 0.23 | 0.99 |
| AA_DQB1_71_32740624_K | -0.34 | 0.18 | 6.18E-02 | 0.23 | 0.99 |
| AA_DQB1_55_32740672_L | -0.34 | 0.18 | 6.18E-02 | 0.23 | 0.99 |
| AA_DQB1_52_32740681 | -0.34 | 0.18 | 6.18E-02 | 0.23 | 0.99 |
| AA_DQB1_47_32740696 | -0.34 | 0.18 | 6.18E-02 | 0.23 | 0.99 |
| AA_DQB1_46_32740699 | -0.34 | 0.18 | 6.18E-02 | 0.23 | 0.99 |
| AA_DQB1_37_32740726_I | -0.34 | 0.18 | 6.18E-02 | 0.23 | 0.99 |
| AA_DQB1_30_32740747_S | -0.34 | 0.18 | 6.18E-02 | 0.23 | 0.99 |
| AA_DQB1_28_32740753 | -0.34 | 0.18 | 6.18E-02 | 0.23 | 0.99 |
| AA_DQB1_-10_32742295_S | -0.34 | 0.18 | 6.18E-02 | 0.23 | 0.98 |
| AA_DRB1_13_32660109_RY | -0.32 | 0.17 | 6.18E-02 | 0.23 | 0.99 |
| AA_DRB1_11_32660115_PG | -0.32 | 0.17 | 6.18E-02 | 0.23 | 0.99 |
| AA_DRB1_13_32660109_R | -0.40 | 0.21 | 6.23E-02 | 0.23 | 0.99 |
| AA_DRB1_11_32660115_P | -0.40 | 0.21 | 6.23E-02 | 0.23 | 0.99 |
| AA_A_65_30018705 | 0.45 | 0.24 | 6.33E-02 | 0.23 | 0.99 |
| AA_A_99_30019048_F | 0.45 | 0.24 | 6.33E-02 | 0.23 | 0.98 |
| AA_A_99_30019048_Y | 0.45 | 0.24 | 6.33E-02 | 0.23 | 0.99 |
| HLA_DQB1_02 | -0.34 | 0.18 | 6.35E-02 | 0.23 | 0.97 |
| AA_B_180_31431931_E | -0.30 | 0.16 | 6.45E-02 | 0.23 | 0.98 |
| AA_B_180_31431931_Q | -0.30 | 0.16 | 6.45E-02 | 0.23 | 0.98 |
| AA_B_177_31431940_D | -0.30 | 0.16 | 6.45E-02 | 0.23 | 0.98 |
| AA_B_177_31431940_E | -0.30 | 0.16 | 6.45E-02 | 0.23 | 0.98 |
| HLA_DQB1_0301 | 0.36 | 0.20 | 6.51E-02 | 0.23 | 0.91 |
| AA_DQB1_45_32740702 | 0.36 | 0.20 | 6.56E-02 | 0.23 | 0.92 |
| AA_C_152_31346921_E | -0.29 | 0.16 | 6.64E-02 | 0.23 | 0.99 |
| HLA_C_0701 | -0.38 | 0.21 | 6.98E-02 | 0.24 | 0.99 |
| AA_C_90_31347357 | -0.28 | 0.15 | 7.03E-02 | 0.24 | 0.99 |
| AA_C_184_31346238_PR | -0.29 | 0.16 | 7.13E-02 | 0.24 | 0.99 |
| AA_C_147_31346936 | -0.29 | 0.16 | 7.13E-02 | 0.24 | 0.99 |
| AA_C_-17_31347805_A | -0.29 | 0.16 | 7.13E-02 | 0.24 | 0.99 |
| HLA_DQB1_0302 | 0.48 | 0.27 | 7.16E-02 | 0.24 | 0.97 |
| AA_DRB1_-24_32665481_L | 0.35 | 0.19 | 7.20E-02 | 0.24 | 0.96 |
| AA_A_282_30020277_V | 0.44 | 0.24 | 7.32E-02 | 0.24 | 0.99 |
| AA_A_311_30020364_N | 0.44 | 0.24 | 7.32E-02 | 0.24 | 0.99 |
| AA_A_282_30020277_I | 0.44 | 0.24 | 7.32E-02 | 0.24 | 0.99 |
| AA_A_311_30020364_K | 0.44 | 0.24 | 7.32E-02 | 0.24 | 0.99 |
| AA_DRB1_26_32660070_L | 0.39 | 0.22 | 7.35E-02 | 0.24 | 0.98 |

BETA: coefficient of the linear regression for association; SE: standard error of BETA; P: the p-value for the association analysis of the minor allele; Q: the *q*-value gives the expected positive false discovery rate

**Supplementary table 3: Frequencies in three reference cohorts and in the GWAS1 and GWAS2 groups, of the *HLA* variants exhibiting a FDR < 0.25 for the continuous trait analyses.**

| **Analysis** | **HLA protein, position, amino-acid(s)** | **Freq reference France (Rennes) cohort (N=200)** | **Freq reference France (west) cohort (N=100)** | **Freq reference Germany (Essen) cohort (N=174)** | **Freq GWAS1 (N=772)** | **Freq GWAS2 (N=1080)** |
| --- | --- | --- | --- | --- | --- | --- |
| Continous GWAS1 | DRB1, 37, tyrosine and leucine | 31.70% | NA | 29.90% | 33.35% | 33.55% |
|  | DRB1, 71, arginine | 44% | NA | 47.60% | 50.06% | 45.17% |
|  | DQB1, 26, leucine | 65.70% | 50.79% | 56.50% | 68.80% | 57.80% |
|  | DRB1, 67, phenylalanine | 11.90% | NA | 16.50% | 16.65% | 18.48% |
| Continous  meta-analysis  (GWAS1, GWAS2) | DRB1, 74, alanine and leucine | 67.00% | NA | 74.40% | 70.27% | 71.19% |
|  | DRB1, 74, arginine and glutamic acid | 15.20% | NA | 15.30% | 16.13% | 16.31% |
|  | *DQB1*02* | NA | 17.23% | 19.30% | 21.70% | 20.57% |
|  | DQB1, 74, alanine | NA | 17.23% | 19.30% | 21.70% | 20.57% |
|  | DQB1, 71, lysine | NA | 17.23% | 19.30% | 21.70% | 20.57% |
|  | DQB1, 55, leucine | NA | 17.23% | 19.30% | 21.70% | 20.57% |
|  | DQB1, 52, leucine | NA | 17.23% | 19.30% | 21.70% | 20.57% |
|  | DQB1, 47, phenylalanine | NA | 17.23% | 19.30% | 21.70% | 20.57% |
|  | DQB1, 46, glutamic acid | NA | 17.23% | 19.30% | 21.70% | 20.57% |
|  | DQB1, 37, isoleucine | NA | 17.23% | 19.30% | 21.70% | 20.57% |
|  | DQB1, 30, serine | NA | 17.23% | 19.30% | 21.70% | 20.57% |
|  | DQB1, 28, serine | NA | 17.23% | 19.30% | 21.70% | 20.57% |
|  | DQB1, -10, serine | NA | 17.23% | 19.30% | 21.70% | 20.57% |

We chose two populations from France (France, Rennes, N=200; France, west, N=100) and one from Germany (Germany, Essen, N=174) with alleles given in at least four digits the Allele frequency net database. For the amino-acid frequencies we used the tool of translation from Allele frequency net database

**Supplementary table 4: Linkage disequilibrium (D’ and *r*^2^) in GWAS1 (N=847) and GWAS2 (N=1292), between the principal associated variants.**

| **D'** | | | | | | | |
| --- | --- | --- | --- | --- | --- | --- | --- |
| **GWAS1** | | | | | | | |
|  | **(1)** | **(2)** | **(3)** | **(4)** | **(5)** | **(6)** | **(7)** |
| **AA_DRB1_74_32659926_AL (1)** | / | **1** | 0.218 | 0.897 | 0.877 | **0.996** | 0.724 |
| **AA_DRB1_74_32659926_RE (2)** | **1** | / | 0.491 | 0.869 | 0.794 | 0.659 | 0.532 |
| **AA_DRB1_71_32659935_R (3)** | 0.218 | 0.491 | / | 0.88 | 0.262 | 0.053 | 0.551 |
| **AA_DRB1_67_32659947_F (4)** | 0.897 | 0.869 | 0.88 | / | 0.78 | **1** | 0.935 |
| **AA_DRB1_37_32660037_YL (5)** | 0.877 | 0.794 | 0.262 | 0.78 | / | **1** | 0.513 |
| **HLA_DQB1_02 (6)** | **0.996** | 0.659 | 0.053 | **1** | **1** | / | **1** |
| **AA_DQB1_26_32740759_L (7)** | 0.724 | 0.532 | 0.551 | 0.935 | 0.513 | **1** | / |
| **GWAS2** | | | | | | | |
|  | **(1)** | **(2)** | **(3)** | **(4)** | **(5)** | **(6)** | **(7)** |
| **AA_DRB1_74_32659926_AL (1)** | / | **0.996** | 0.258 | 0.801 | **0.903** | **0.967** | 0.632 |
| **AA_DRB1_74_32659926_RE (2)** | **0.996** | / | 0.308 | 0.604 | 0.813 | 0.525 | 0.333 |
| **AA_DRB1_71_32659935_R (3)** | 0.258 | 0.308 | / | 0.936 | 0.28 | 0.03 | 0.662 |
| **AA_DRB1_67_32659947_F (4)** | 0.801 | 0.604 | 0.936 | / | 0.744 | 0.87 | 0.86 |
| **AA_DRB1_37_32660037_YL (5)** | **0.903** | 0.813 | 0.28 | 0.744 | / | **0.914** | 0.438 |
| **HLA_DQB1_02 (6)** | **0.967** | 0.525 | 0.03 | 0.87 | **0.914** | / | **1** |
| **AA_DQB1_26_32740759_L (7)** | 0.632 | 0.333 | 0.662 | 0.86 | 0.438 | **1** | / |
| ***r*^2^** | | | | | | | |
| **GWAS1** | | | | | | | |
|  | **(1)** | **(2)** | **(3)** | **(4)** | **(5)** | **(6)** | **(7)** |
| **AA_DRB1_74_32659926_AL (1)** | / | 0.457 | 0.02 | 0.068 | 0.164 | **0.65** | 0.154 |
| **AA_DRB1_74_32659926_RE (2)** | 0.457 | / | 0.046 | 0.029 | 0.061 | 0.302 | 0.038 |
| **AA_DRB1_71_32659935_R (3)** | 0.02 | 0.046 | / | 0.156 | 0.035 | 0.001 | 0.212 |
| **AA_DRB1_67_32659947_F (4)** | 0.068 | 0.029 | 0.156 | / | 0.241 | 0.055 | 0.251 |
| **AA_DRB1_37_32660037_YL (5)** | 0.164 | 0.061 | 0.035 | 0.241 | / | 0.14 | 0.191 |
| **HLA_DQB1_02 (6)** | **0.65** | 0.302 | 0.001 | 0.055 | 0.14 | / | 0.192 |
| **AA_DQB1_26_32740759_L (7)** | 0.154 | 0.038 | 0.212 | 0.251 | 0.191 | 0.14 | / |
| **GWAS2** | | | | | | | |
|  | **(1)** | **(2)** | **(3)** | **(4)** | **(5)** | **(6)** | **(7)** |
| **AA_DRB1_74_32659926_AL (1)** | / | 0.494 | 0.021 | 0.063 | 0.173 | **0.579** | 0.123 |
| **AA_DRB1_74_32659926_RE (2)** | 0.494 | / | 0.025 | 0.018 | 0.07 | 0.221 | 0.017 |
| **AA_DRB1_71_32659935_R (3)** | 0.021 | 0.025 | / | 0.165 | 0.032 | 0 | 0.258 |
| **AA_DRB1_67_32659947_F (4)** | 0.063 | 0.018 | 0.165 | / | 0.255 | 0.046 | 0.235 |
| **AA_DRB1_37_32660037_YL (5)** | 0.173 | 0.07 | 0.032 | 0.255 | / | 0.11 | 0.133 |
| **HLA_DQB1_02 (6)** | **0.579** | 0.221 | 0 | 0.046 | 0.11 | / | 0.19 |
| **AA_DQB1_26_32740759_L (7)** | 0.123 | 0.017 | 0.258 | 0.235 | 0.133 | 0.19 | / |

D’: bold > 0.9 and red > 0.9 between HLA-DRB1 and HLA-DQB1 alleles; for *r*^2^: bold > 0.5 and red > between HLA-DRB1 and HLA-DQB1

**Supplementary table 5: Comparison in the recessive model between the non-responders and responders of GWAS1 and GWAS2 for the HLA-DRB1 A/L allele at position 74.**

| Phenotype | GWAS | N | | Recessive model | | p-value | p-value meta (Q-value) |
| --- | --- | --- | --- | --- | --- | --- | --- |
|  |  | Non- resp | Resp | Non-resp (%) | Resp (%) |  |  |
| Dichotomous | GWAS1 | 186 | 418 | 42.47 | 53.83 | 0.01 | 0.0005  (0.47) |
|  | GWAS2 | 201 | 685 | 42.29 | 53.14 | 0.02 |  |
| Extreme | GWAS1 | 186 | 275 | 21.05 | 31.25 | 0.02 | 9.531e-05  (0.09) |
|  | GWAS2 | 201 | 362 | 35.29 | 41.18 | 0.002 |  |

The groups of Li non-responders and Li responders were compared in the dichotomous phenotype (Alda ≤ 3 and Alda ≥ 7) and in the extreme phenotype (Alda ≤ 3 and Alda ≥ 9) in the recessive model for patients carrying only the A/L alleles versus the others. The p-value (Q-value) are the values obtained by performing the random-effects-model meta-analysis of GWAS1 and GWAS2 groups. The recessive effect is very clear by observing the extreme phenotype with a q-value at **0.09**. Non-resp = Li non responders; Resp = Li responders. N = number of patients in the group; recessive model = percentage of patients carrying only the A or L allele. P-value meta = p-value obtained from GWAS1 and GWAS2 meta-analysis .
